# Generation of Magnetized Olfactory Ensheathing Cells for Regenerative Studies in the Central and Peripheral Nervous Tissue

**DOI:** 10.3390/ijms140610852

**Published:** 2013-05-24

**Authors:** Cristina Riggio, Sara Nocentini, Maria Pilar Catalayud, Gerardo Fabian Goya, Alfred Cuschieri, Vittoria Raffa, José Antonio del Río

**Affiliations:** 1 Institute of Life Science, Scuola Superiore Sant’Anna, Piazza Martiri della Libertà 33, Pisa 56127, Italy; E-Mails: a.cuschieri@sssup.it (A.C.); vraffa@biologia.unipi.it (V.R.); 2 Molecular and Cellular Neurobiotechnology, Institute for Bioengineering of Catalonia (IBEC), Barcelona Science Park, Baldiri Reixac 15-21, Barcelona 08028, Spain; E-Mails: snocentini@ibec.pcb.ub.es (S.N.); jadelrio@ibecbarcelona.eu (J.A.R.); 3 Department of Cell Biology, Faculty of Biology, University of Barcelona, Diagonal 643, Barcelona 08028, Spain; 4 Networked Biomedical Research Center for Neurodegenerative Diseases (CIBERNED), Barcelona 08028, Spain; 5 Nanoscience Institute of Aragón, University of Zaragoza, Mariano Esquillor, Zaragoza 50018, Spain; E-Mails: pilarcs@unizar.es (M.P.C.); goya@unizar.es (G.F.G.); 6 Department of Biology, University of Pisa, Via Luca Ghini 5, Pisa 56126, Italy

**Keywords:** nerve regeneration, olfactory ensheathing cell, magnetic nanoparticle, organotypic culture

## Abstract

As olfactory receptor axons grow from the peripheral to the central nervous system (CNS) aided by olfactory ensheathing cells (OECs), the transplantation of OECs has been suggested as a plausible therapy for spinal cord lesions. The problem with this hypothesis is that OECs do not represent a single homogeneous entity, but, instead, a functionally heterogeneous population that exhibits a variety of responses, including adhesion and repulsion during cell-matrix interactions. Some studies report that the migratory properties of OECs are compromised by inhibitory molecules and potentiated by chemical gradients. In this paper, we report a system based on modified OECs carrying magnetic nanoparticles as a proof of concept experiment enabling specific studies aimed at exploring the potential of OECs in the treatment of spinal cord injuries. Our studies have confirmed that magnetized OECs (i) survive well without exhibiting stress-associated cellular responses; (ii) *in vitro*, their migration can be modulated by magnetic fields; and (iii) their transplantation in organotypic slices of spinal cord and peripheral nerve showed positive integration in the model. Altogether, these findings indicate the therapeutic potential of magnetized OECs for CNS injuries.

## 1. Introduction

The elongation of newly generated olfactory receptor axons in the adult from the olfactory mucosa towards the central nervous system (CNS) is attributed to the supporting properties of olfactory ensheating cells (OECs), which ensheath and guide these axons [1,2]. Given their axon growth-promoting properties, OECs (natural or modified) have been transplanted into the injured spinal cord to promote axonal regeneration and functional recovery [3–8]. Indeed, OEC transplantation has emerged as a promising therapy for spinal cord injuries [9,10]. It has been suggested that OECs provide a higher regenerative potential than Schwann cells [11–13]. This property has been attributed to the greater migratory potential of OECs [14]. However, different migratory properties have been reported after OEC transplantation in injured CNS [15,16]. For example, Lu *et al*. did not observe special migratory properties following their implantation in spinal cord lesion, and some of the transplanted cells migrated far from the lesion site [17]. In the injured spinal cord, damaged axons and transplanted cells are exposed to a changing environment with a large variety of growth inhibitory molecules located in the meningo-glial scar and adjacent spinal cord regions [18,19]. OEC migration is modulated by neurotrophins, such as the glial cell-derived neurotrophic factor (GDNF), or chemicals, such as lysophosphatidic acid (LPA) [20–22]. These observations raise the notion that OECs are able to respond to a large array of molecules, which affect their migration, including inhibitory molecules, like Nogo-A [23]. Hence, knowledge of the mechanisms involved in inhibiting OEC migration is useful in the development of appropriate therapeutic strategies. It has already been established in rats that OECs labelled with magnetic nanoparticles (MNPs) can be tracked *in vivo* by MRI to determine their migration details in normal and injured spinal cords, including the possibility that OECs can cross a complete spinal cord injury zone [24]. MNPs are largely employed in biomedicine. The use of MNP has been established for many clinical diagnostic/therapeutic uses, e.g., MRI contrast agents in magnetic resonance imaging [25], for cell tracking via MRI [26], magnetic hyperthermia [27], gene therapy [28], vectors for drug delivery [29], *etc*.

The idea to use MNPs for magnetic labeling of cells has been already proposed in the literature [30] and also proven by our group. When mammalian cells are cultured in a MNP modified medium, MNPs interact with the cells and, following an exposition to a magnetic field, they are able to move cells in the direction imposed by the external magnetic field. By incubating neuroblastoma cell lines with magnetic carbon nanotubes (CNTs), under the effect of a permanent dipole magnet, a progressive displacement of cells toward the more intensive magnetic fields was observed, and results suggested that cell displacement preferentially occurs during cell duplications [31,32]. In another study, mesenchymal stem cells (MSCs) were cultured in a CNT containing medium and, as a result of cell interaction with CNTs, the application of a magnetic field enabled shepherding of MSCs to the desired location *in vivo*. Specifically, when CNT labelled MSCs were injected in the portal vein of rats, we were able to significantly increase the localization of MSCs within the liver, with a consequent reduction of their migration to other organs [33]. More recently, we synthesized iron oxide MNPs with high saturation magnetization and tested them for cellular actuation of a neuroblastoma cell line through external magnetic forces. Results from scanning electron microscopy (SEM) and dual-beam SEM/focused ion beam (FIB) microscopy demonstrated the effective MNP internalization in large amounts. Since the magnetic force exerted on a single cell is proportional to the magnetic moment of the incorporated MNPs for a given magnetic field value (and its spatial derivative), this MNP internalization resulted in a high capability to induce cell migration under the effect of the magnetic source [34].

In an *ex vivo* model involving organotypic culture, the present study explored the utility of magnetizing OECs by labelling them with homemade MNPs, as this would enable precise shepherding of the transplanted cells to a nerve lesion. The results indicate that the labelling does not harm the OECs, and that migration of the magnetized OECs can be directed precisely by a magnetic field. Lastly, magnetized OECs can be transplanted in organotypic slices of spinal cord and peripheral nerve. In these organotypic co-cultures, magnetized OECs survive and are able to integrate within the tissue. Altogether, the method described represents a new approach for controlling the migration of the OECs *in vitro* and appears useful for future studies aimed at effective regeneration of spinal cord injuries.

## 2. Results

### 2.1. Magnetization of OECs via MNPs

**Figure 1:**
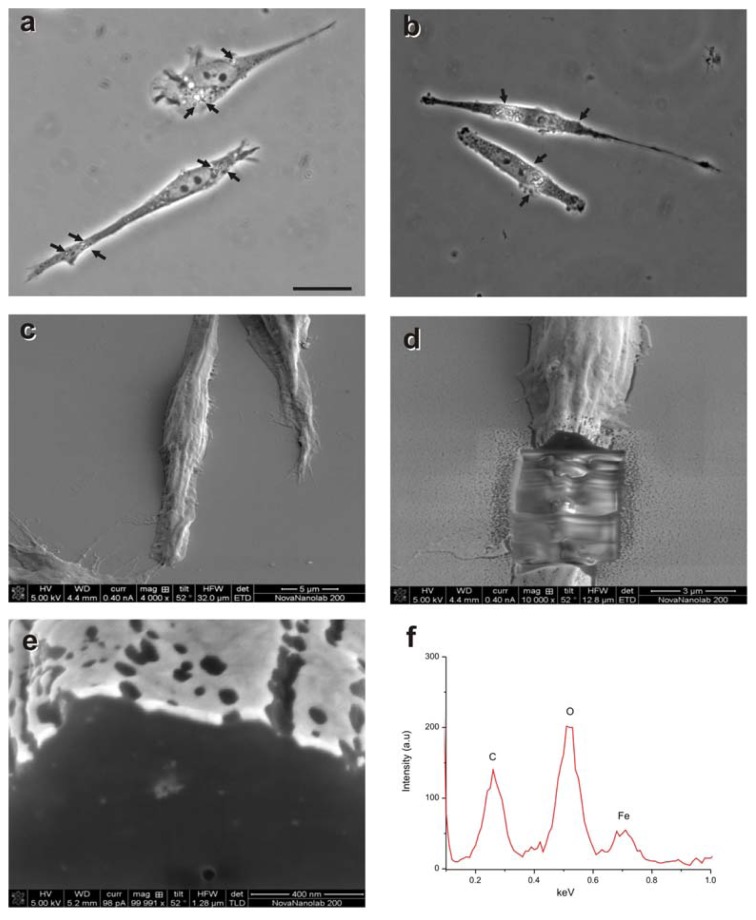
(**a**,**b**) Light microscopy photomicrographs of olfactory ensheathing cells (OECs) treated with 10 μg/mL (**a**) or 25 μg/mL (**b**) of MNPs (M-OECs) (arrows in a and b); (**c**,**d**) dual beam SEM/FIB images of M-OECs treated with 10 μg/mL of MNPs. The internalization of the MNPs can be seen in the cross section of single OEC cells (light grey spots in **e**) and confirmed by the Fe-content from EDS analysis of these areas (spectral analysis in **f**).

OECs were cultured with MNPs (M-OECs) for 24 h and analyzed by light microscopy (Figure 1a,b) and electron microscopy (Figure 1c,d). Few particles could be detected on the cell surface, whereas clusters of particles were found inside cell cytoplasm. EDS (energy-dispersive X-ray spectroscopy) spectra clearly showed that these agglomerates are composed of iron, reflecting the intracellular presence of the MNPs (Figure 1e,f) and confirming the efficient labelling process of the OECs.

### 2.2. M-OECs Viability

**Figure 2:**
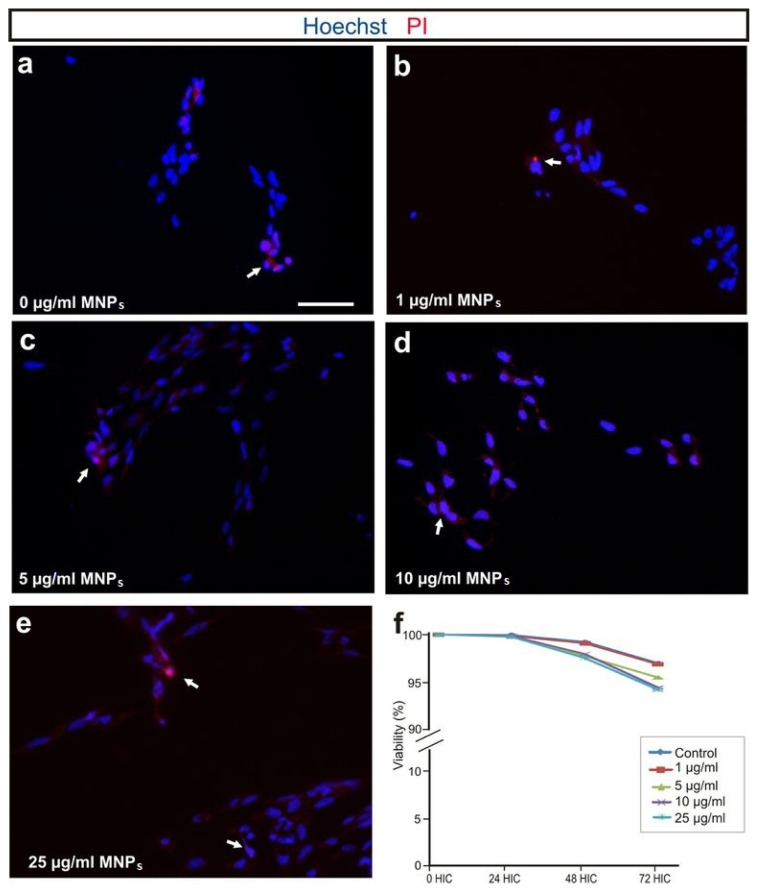
(**a**–**e**) Fluorescent microscopy of cells treated with MNPs 0, 1, 5, 10 and 25 μg/mL, respectively. Blue: Hoechst nuclear staining. Red: PI staining (white arrows show PI positive cells); (**f**) PI staining via flow cytometry of cells treated for 72 h with MNPs (1–25 μg/mL) (*p* > 0.05, ANOVA).

**Figure 3:**
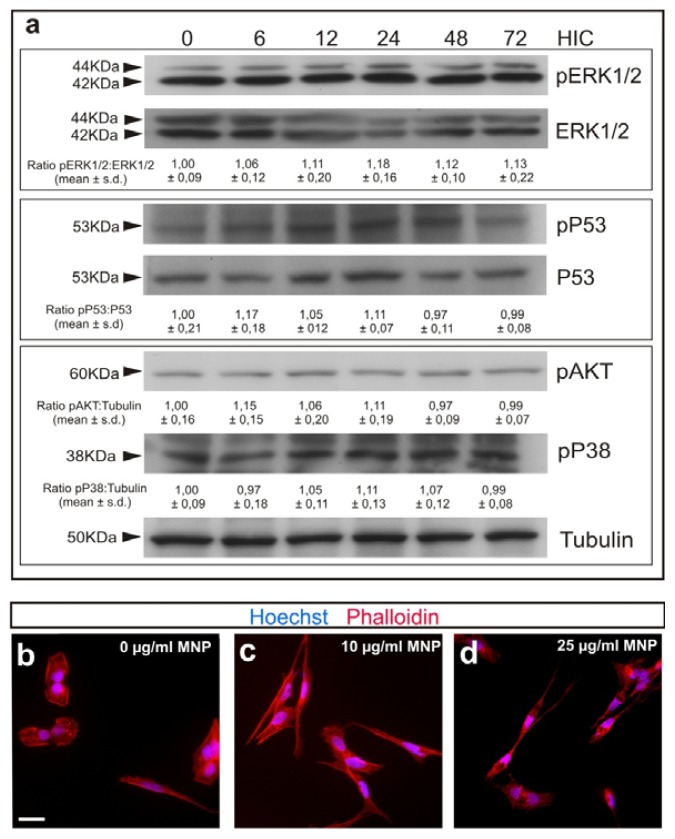
(**a**) Western blot analysis for M-OECs treated with MNPs (10 μg/mL) for 0, 6, 12, 24, 48 and 72 h in culture (HIC). Notice that activation levels of pERK1/2, pAKT, pP38 and pP53 are unchanged after MNP treatment. *p* > 0.1, ANOVA. (**b**–**d**) Phalloidin (red) and Hoechst (blue) staining for cells treated with 0, 10 and 25 μg/mL of MNPs for 24 h (b,c and d, respectively).

Cell viability of M-OECs was tested in both a time- and dose-dependent manner by using propidium iodide (PI) dye exclusion assay. Flow cytometer analysis showed that the treatment induces a negligible toxicity after 72 h of treatment with all the concentrations of MNPs tested. In particular, at the highest concentration tested (25 μg/mL), the viability was found ~94.25% ± 3.12%, and not far from the control (~97.02% ± 5.05%) (Figure 2f). These results were confirmed by PI staining via fluorescent microscopy (Figure 2a–e); cells treated with MNPs exhibited permeability to PI similar to the control, except for 25 μg/mL, where few red spots are noticeable. Immunoblot analysis was performed to document any differences in the expression of proteins involved in cell replication (AKT, protein kinase B), apoptosis (P53) and cell death (ERK, extracellular-signal-regulated kinases) between the control and the cell treated with 10 μg/mL of MNPs over time. At any time tested, no differences were found in the protein expression and their phosphorylation level confirming the negligible toxicity induced on OECs by MNP labelling (Figure 3a).

The cytoskeleton of OECs is the main target during the inhibition of cell migration [35]. Actin filaments staining confirmed that the labelling with MNPs did not alter cell morphology or induce cytoskeleton rearrangement (Figure 3b–d). Overall, these results confirm the negligible cytotoxic profile of MNP labelling.

### 2.3. M-OECs Movement toward a Magnetic Source

**Figure 4:**
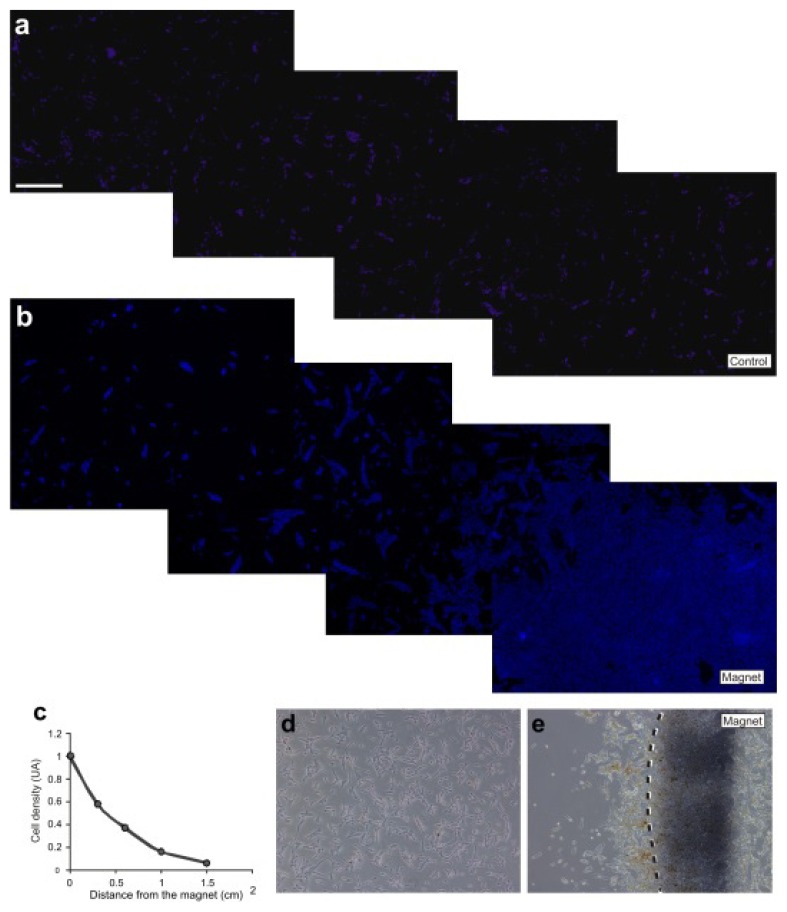
(**a**) Hoechst staining of M-OECs treated with MNPs (10 μg/mL); (**b**) Hoechst staining of M-OECs treated with MNPs (10 μg/mL); 8 h after the seeding, the magnet was placed inside the 3,5 mm ø petri dish for 12 h, when the images were acquired. (**c**) Quantification of cell density (*y*-axis) *vs*. distance from the magnet (*x*-axis) in the experiments. Note the increased number of M-OECs close to the magnet. (**d**,**e**) White field of a and b, respectively.

When magnetically labelled cells are exposed to a non-homogeneous static magnetic field, the magnetic force exerted on the incorporated MNPs should result in a preferential growth and cell migration towards the magnetic source. Cell movement of M-OECs towards the magnetic source was monitored by fluorescent microscopy (nuclear staining). A magnet was attached to the cover of the Petri dish, and cell density was measured after 12 h as a function of the distance from the magnet (Figure 4). As expected, cell density increases exponentially with the decrease of the distance from the magnet, indicating that the magnetic field can actuate the labelled cells and pull them towards the magnetic poles, in agreement with our previous findings with different cellular models [33,36].

### 2.4. M-OECs Survive in an Organotypic Co-Culture of Peripheral Nerve and Spinal Cord

OECs have been used in numerous studies of axon regeneration (see introduction for details). Some of the studies determined both the poor survival of transplanted OECs and migratory deficits of these cells [9].

**Figure 5:**
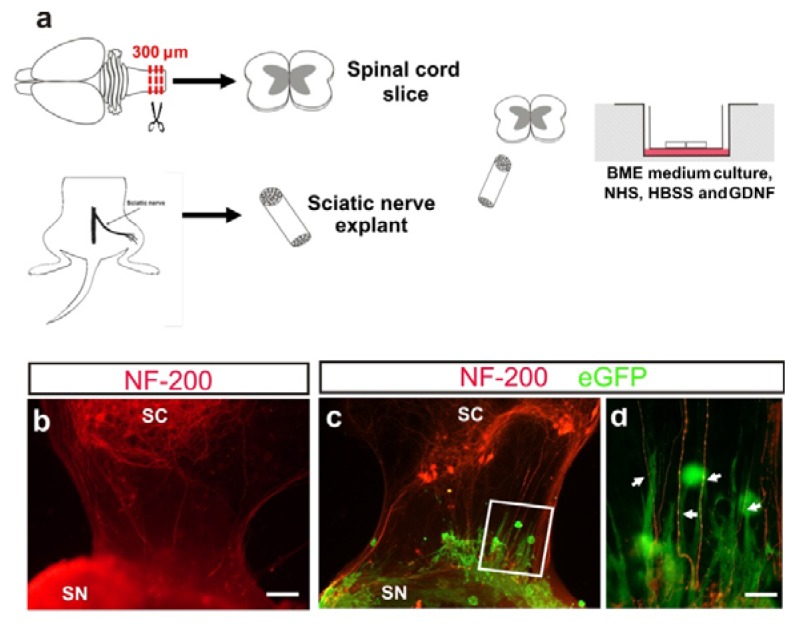
(**a**) Representation of spinal cord and sciatic nerve co-culture (see material and methods for details). (**b**) Fluorescence photomicrograph illustrating an example of organotypic co-cultures of spinal cord (SC) and peripheral nerve (SN) in absence of M-OECs. After seven days *in vitro* (DIV), axons from motor neurons (red) reach and innervate the sciatic nerve explant. (**c**,**d**) Example of the morphology and behavior of M-OECs after transplantation in the organotypic slices. Numerous eGFP-positive cells can be seen, and some of them oriented parallel to the motor neuron axons (arrows in d). Scale bars: b, c = 150 μm, d = 50 μm.

The olfactory ensheathing cell line used in 3D co-cultures experiments were obtained by lentiviral infection with eGFP-expressing lentivirus (see [37] for details). The model used consists of spinal cord slices co-cultured with a sciatic nerve graft for ventral root reconstruction (Figure 5a) (see also [38] for details).

Immunocytochemistry performed on organotypic co-cultures of this peripheral nerve regeneration model revealed that after seven days *in vitro*, motor neuron axons (NF-200 positive) were able to reach the sciatic nerve explants (Figure 5b). M-OECs were added to the model in order to assess the survival and the behavior of grafted cells. We observed the presence of M-OECs (eGFP-positive) after seven days of incubation in close distribution to NF-200 positive fibers (Figure 5c,d). In addition, numerous M-OECs were oriented parallel to NF-200 positive axons (Figure 5d). These data indicate that M-OECs were able to integrate into organotypic culture with some of them in close relationship with growing axons. Thus, the behavior of M-OECs is similar to those reported in other *in vitro* models using OECs [39].

## 3. Discussion

Cell transplantation has been used as therapy after CNS lesions. Since nerve regeneration can be promoted by supplying supportive ECM components and NFs and cell adhesion molecules, cells (e.g., Schwann cells) are effective and appropriate vehicles for supplying these factors [40–42]. Thus, normal or engineered cells have been largely used in several lesions in both central and peripheral nervous systems (e.g., [9,43,44]). However, as cell survival and migration may be compromised when cells are transplanted in an inhibitory environment [41,45], measures to enhance and direct transplanted cells are needed. In the present study, we propose a cell therapy strategy based on magnetized OECs [46,47]. Glial cells (*i.e*., Schwann cells) and macrophages support regeneration by clearing debris and secreting neurotrophic factors to aid axonal outgrowth [42]. In addition, OECs, a special type of glia cells that share phenotypic similarities to Schwann cells, are being extensively investigated as transplants to support nerve regeneration [48,49]. OECs support and guide the growth of olfactory axons and ensheath the bundles of olfactory nerves that extend from the olfactory epithelium to the olfactory bulb (e.g., [45]). Based on these features together with the capacity of OECs to express a number of neurotrophic factors [50], these cells are considered as candidate donor cells for axonal injuries and demyelinating diseases (e.g., [9,51]).

The migratory properties of the OECs are crucial for neural regeneration [35]. Several studies have reported that the migrating behavior of OECs varies and falls into two subpopulations: Schwann cell-like and the astrocyte-like morphology [45,52]. Irrespective of type, it is possible to magnetize OECs with MNPs and, thus, control their varied migratory behavior into “controlled shepherding” by magnetic fields. In order to confirm that MNP-labelled cells move towards a magnetic source, we performed cell migration experiments and demonstrated that magnetic labelling induces controlled and efficient cell migration under the influence of magnetic field gradients. These results are in agreement with our previous findings on MNP-labelled human neuroblastoma cells, showing that incorporation of MNPs into cells can render them suitable for magnetic actuation by external magnetic fields. Specifically, migration experiments under external magnetic fields confirmed that MNPs produced in our lab can effectively actuate the cells, inducing directional measurable migration towards predefined targets more effectively than commercial nanoparticles (e.g., fluidMAG-ARA supplied by Chemicell) [34].

In the present study, we demonstrated that the magnetization protocol we have developed does not alter biological features of OECs, which interacted strongly with MNPs with internalization of the MNPs demonstrated by electron microscopy. Western blot analysis revealed that MNP labelling does not induce activation (or phosphorylation) of proteins involved in cell replication, apoptosis and cell death (Figure 3a). The same results were obtained by both quantitative and qualitative PI dye exclusion assays, which confirmed cell viability exceeding 90% at all concentrations tested. Likewise, there was no cytoskeletal reorganization demonstrated with actin staining (Figure 3b–d).

In this study, we analyzed the survival of M-OECs in an organotypic model of spinal cord and peripheral nerve. Although *in vitro* transplantation of OECs and spinal cord has been reported [39], to our knowledge, this is the first report transplanting magnetized OECs in 3D co-cultures of spinal cord and peripheral nerve. Organotypic cultures are biochemically and physiologically similar to *in vivo* tissue and are very useful for studies on neural regeneration and drug delivery [53,54]. Several reports [55] have confirmed that nerve regeneration could be reproduced *in vitro* by using an organotypic model made of spinal cord slices and ulnar nerve [38]. In the present study, spinal cord slices and sciatic nerve explants from neonatal mice were cultured for seven days. Sciatic nerve was placed in front of ventral roots to allow motor neurons to innervate the sciatic nerve. After seven days of incubation, axons from motor neurons of the ventral horn of the spinal cord reached sciatic nerve. Hence, this system represents a good *in vitro* model for studying nerve regeneration of a peripheral nerve.

The behavior of M-OECs entrapped in Matrigel™ was tested by placement on the membrane used for organotypic co-cultures, with the regeneration process being monitored by staining motor neurons with NF-200 antibody (see Experimental section for details). This regeneration model showed that axons from the motor neurons of the ventral root of spinal cord re-innervated the sciatic nerve by grossing the 7 mm gap (site of lesion) between the two explants. The transplantation of M-OECs (Figure 5) reported a good survival in these conditions (at least after seven days), with behavior resembling those reported by untreated OECs in similar models [39].

## 4. Experimental Section

### 4.1. MNP Synthesis

The synthesis protocol was based on the oxidative hydrolysis method, *i.e*., the precipitation of an iron salt (FeSO_4_) in basic media (NaOH) with a mild oxidant. In a typical synthesis, a mixture of 1.364 g of KNO_3_ and 0.486 g of NaOH was dissolved in 135 mL of distilled water in a three-necked flask bubbled with N_2_. Then, 15 mL of 0.01 M H_2_SO_4_ solution containing 0.308 g of FeSO_4_·7H_2_O (previously flowed with N_2_ for 2 h) and polyethyleneimine (PEI) was added dropwise under constant stirring [56]. When the precipitation was completed, nitrogen was allowed to pass for another 5 min, and the suspension with black precipitate was held at 90 °C for 24 h under N_2_. Afterward, the solution was cooled at room temperature with an ice bath, and the solid was separated by magnetic decantation and washed several times with distilled water. All reagents were commercially available and used as received without further purification. Iron (II) sulfate heptahydrate (FeSO_4_·7H_2_O), sodium hydroxide (NaOH), potassium nitrate (KNO_3_), sulfuric acid (H_2_SO_4_) and polyethylenimine (PEI, MW = 25 kDa) were obtained from Sigma Aldrich.

### 4.2. OEC Cultures

The immortalized clonal OEC cell line, TEG3, was maintained in ME10: DMEM–F12 (Invitrogen, Carlsbad, CA, USA) supplemented with 10% bovine calf serum (SAFC Biosciences, Lanexa, VA, USA), 20 μg/mL pituitary extract (Invitrogen, Carlsbad, CA, USA), 2 μM forskolin (Sigma-Aldrich, St. Louis, MO, USA), 1% penicillin-streptomycin and 1% Fungizone (Invitrogen, Carlsbad, CA, USA).

### 4.3. Spinal Cord (SC) Isolation and Sciatic Nerve(SN) Isolation

Mouse pups (P5-P6) were decapitated cleanly outside the sterile area by cutting with large scissors at the foramen magnum. The backbone was approached through a midline laparotomy; the bone cut with scissors and the lumbar region isolated, removed and put in cold PBS 0.1 M with 6.5 mg/mL glucose. Using a dissection microscope, the bone was carefully peeled off, taking care to remove also the meninges. The isolated lumbar spinal cord was put in the tissue chopper and cut into 350 μm slices, which were placed in incubation medium for 30 min and then seeded over a Millicell transwell. The incubation medium consisted of basal medium Eagle (BME) (50%), horse serum (25%), Hanks (2.5%), glucose (1%), glutamine (1%), GDNF (10 ng/mL) and water. After removing the excess of medium from the insert and placing the insert in the 6 well plates, slices were incubated at 37 °C. The sciatic nerve was harvested from an adult mouse after sacrifice. Again, the dissection microscope was used to expose the sciatic nerve, which was harvested and put in the incubation medium. The sciatic nerve was co-cultured with the spinal cord slice, by placing in front of the ventral root of the spinal cord, leaving a small gap in which M-OECs were injected in Matrigel™.

### 4.4. PI Dye Exclusion Assay through Fluorescence Microscopy

Cells (5 × 10^4^) were seeded in 24 well plates. 24 h later, cells were incubated for 72 h in modified cell culture medium. PI was added at 2.5 μg/mL to each well and let to incubate for 5 min at room temperature (RT). Cells were washed two times with PBS and then fixed with paraformaldehyde 4% for 10 min at RT. Cells were washed again 2 times with PBS and then re-suspended in PBS. Hoechst 1 μM was added and incubated for 15 min at RT. Cells were washed three times with PBS and then analyzed in fluorescence.

### 4.5. PI Dye Exclusion Assay through Flow Cytometry

Cells (5 × 10^4^) were seeded onto coverslips in 24 well plates in ME10 medium. Twenty-four hours later, cells were incubated for 72 h in the presence of different concentrations of nanoparticles (1, 5, 10, 25 μg/mL) in the medium. PI was added at 2.5 μg/mL to each well and incubated for 5 min at room temperature (RT). Cells were then fixed in 4% buffered paraformaldehyde, rinsed in 0.1 M PBS, stained with 0.1 μM Hoechst diluted in 0.1 M PBS for 10 min, rinsed in 0.1 M PBS and mounted on Fluoromount™ (Vector Labs, Burlingame, CA, USA). Alternatively, the percentage of nonviable cells was measured using a modification of the method described by [57]. Five thousand cells were plated into a 6-well plate with 2.5 mL of ME10 medium. Different concentrations of magnetic nanoparticles (0–50 μg/mL) were added to the cell medium. Baseline fluorescence *F*_1_ was measured 1 h after addition of PI (30 μM) as an index of cell death not related to the treatment. Subsequently, fluorescence readings were taken at different times after the onset of the treatment (24, 48 and 72 h of incubation with MNPs). At the end of the experiment, the cells were permeabilized for 10 min with 500 μM digitonin at 37 °C to obtain the maximum fluorescence corresponding to 100% of cell death (*F*_max_). PI fluorescence was measured in 24-well plates using a CytoFluor 2350 scanner (Millipore Corporation, Billerica, MA, USA) with 530 nm excitation (25 nm band pass) and 645 nm (40 nm band pass) emission filters. The percentage of cell death was calculated as follows:

$$\%\;\text{cell death}=(F_{n}-F_{1})\times 100/(F_{\text{max}}-F_{1})$$

where *F_n_* is the fluorescence at any given time.

### 4.6. Immunoblotting Analysis

This was performed to examine the expression level of specific proteins in cells treated with 10 μg/mL of MNPs for 0, 6, 12, 24, 48 and 72 h. The total cell lysate was prepared using a lysis buffer (50 mM Hepes, 1.5 mM MgCl_2_, 150 mM NaCl, 1% Triton 100X, 10% glycerol 10%, 1 mM EGTA and 13 protease inhibitor cocktail (Roche, Basel, Switzerland)), followed by centrifugation and magnetic separation removal. The protein content was determined using the Bio-Rad detergent-compatible assay (BCA) (Bio-Rad, Hercules, CA, USA). Cell extracts (20 μg total protein) were boiled in Laemmli sample buffer at 100 °C for 10 min, subjected to 10% SDS-PAGE and electro-transferred to nitrocellulose membranes (Amersham Biosciences, Amersham, UK). After transfer, the membrane was blocked for 1 h with blocking buffer (5% milk in TBTS); the membrane was incubated overnight at 4 °C with anti-tubulin (1:5000), anti-pERK1/2 and anti-ERK1-2, anti-pAKT, anti-pP38, anti-pP53 and anti-P53, all diluted to 1:1000 (*v*/*v*) in TBTS.

After washing (0.1% Tween 20 in PBS, pH 7.4), the membrane was incubated for 2 h at RT with secondary antibodies diluted to 1:1000 (*v*/*v*). The membranes were visualized using the ECL detecting solution (Millipore Corporation, Billerica, MA, USA) and analyzed using a Syngene imaging analysis system and software (Syngene, Cambridge, UK).

### 4.7. Phalloidin Staining

The cytoskeletal rearrangement of cells was studied by means of actin staining. Cells were seeded in a coverslip, at a concentration of 5·× 10^4^ cells/well and incubated overnight in ME10 medium to allow cell adhesion. MNPs were added in the medium at a concentration of 10 μg/mL. Cells were cultured for 3 days before carrying out F-Actin staining. The culture medium was removed, and the cells were gently washed with PBS at 4 °C and, then, fixed with formaldehyde 4% for 15 min. After washing, cells were permeabilized with 0.1% Triton X-100 in 0.1 M PBS and blocked with 10% normal serum in 0.1 M PBS. Cells were sequentially incubated with Phalloidin-Rhodamine (R415, Invitrogen, Carlsbad, CA, USA) for 1 h, stained for Hoechst for 10 min, rinsed in 0.1 M PBS and mounted on Fluoromount™. The images were analyzed by fluorescent microscopy.

### 4.8. Immunocytochemistry of SC/SN Slice/OEC Co-Cultures

The SC/SN slice/OEC co-cultures were fixed in 4% PFA in PBS for 30 min at RT and washed with Triton 0.7% and PBS with gelatin 0.02% to permeabilize the cultures. Blocking was performed using Triton 0.7%, PBS with gelatin 0.02% and FBS 10% for 5 h 4 °C. Before proceeding with the incubation, the preparations were washed 4 times for 5 min with PBS. The co-cultures were then incubated with Triton 0.7%, PBS with gelatin 0.02% and FBS 5% with NF-200 (1:250) primary antibody for 48 h at 4 °C. After washing again for 5 min with PBS, the co-cultures were incubated with secondary antibody. Membranes with co-cultured SC/SN slice and OECs were cut off and mounted on glass slides with Fluoromount™ aqueous mounting medium and examined using an Olympus BX61 fluorescence microscope.

### 4.9. SEM/FIB Analysis

SEM/FIB cross sectioned cells were performed using SEM INSPECT F50, FEI Company, Hilsboro, OR, USA) and dual-beam FIB/SEM (Nova 200 NanoLab, FEI Company, Hilsboro, OR, USA). SEM images were taken at 5 and 30 kV, with a FEG column, and a combined Ga-based 30 kV (10 pA) ion beam was used to cross-section single cells. These investigations were completed by EDS for chemical analysis.

OECs were grown on coverslips and treated with MNPs (10 μg/mL). After 24 h of incubation, the cells were washed with PBS, fixed and dehydrated. After drying, the samples were sputtered with 10 nm of gold.

### 4.10. Cell Migration Assay

OECs were detached by trypsinization, collected by centrifugation and seed with fresh medium. After 24 h of incubation, 10 μg/mL of MNPs were added and then let to incubate for other 48 h. For the selection of the “magnetized” cell population, cells were trypsinized and subjected to magnetic separation. M-OECs were counted in a Burker chamber and seeded in 3.5 cm Petri dish. After 8 h of incubation, a neodymium cylindric magnet (N12, radius 0.3 length, height 0.8 mm) was attached to the cover of the Petri dish (Figure S1). Cell density was visualized by nuclear staining (Hoechst, Sigma, 33258) after 12 h.

### 4.11. Statistical Analysis

Values are reported as the mean ± standard error of the mean (S.E.M). Statistical significance was assessed by one way analysis of variance (ANOVA).

## 5. Conclusions

In this study we demonstrated that OeC labeled with magnetic nanoparticles can be manipulated by an external magnetic field and show a positive integration in organotypic slices of spinal cord and peripheral nerve.

These results pave the way for *in vivo* use of magnetized OECs and magnetic fields as a tool to localize the OEC-mediated production of therapeutic molecules at the lesion site. In such *in vivo* protocol, M-OECs would be injected at the site of the nerve injury site and magnetic fields (with the appropriate simulation and design of the magnetic field geometry) used to enhance homing and the localization of M-OECs to the injury site. In a previous study, we reported that magnetically labelled cells can be shepherded *in vivo* to a desired location following their intravenous administration and application of a proper magnetic field [33]. Further work is needed to demonstrate the capability to shepherd *in vivo* magnetically labelled OECs to the nerve injury site to promote regeneration by enhancing axon growth across the lesion and functional recovery.

## Supplementary Information
