# Supplementary material for: Generation of Magnetized Olfactory Ensheathing Cells for Regenerative Studies in the Central and Peripheral Nervous Tissue
Source: Int J Mol Sci. 2013 May 24;14(6):10852–68. doi: 10.3390/ijms140610852 (PMC3709706; doi:10.3390/ijms140610852)
Supplement: Supplementary file 1 [file ijms-14-10852-s001.pdf]

Supplementary Information

Figure S1. Experimental set-up for cell movement. (**A**) Cell density in the petri dish before to place the magnet; (**B**) Cell density in the petri dish 12 h later the magnet was put inside.

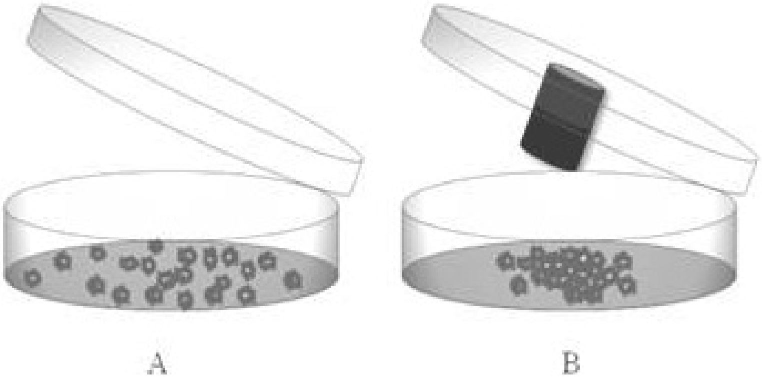

© 2013 by the authors; licensee MDPI, Basel, Switzerland. This article is an open access article distributed under the terms and conditions of the Creative Commons Attribution license (http://creativecommons.org/licenses/by/3.0/).
